# FERTILITY CARE IN LOW AND MIDDLE INCOME COUNTRIES: The landscape of assisted reproductive technology access in India

**DOI:** 10.1530/RAF-24-0079

**Published:** 2024-11-11

**Authors:** Prathima Tholeti, Shubhashree Uppangala, Guruprasad Kalthur, Satish Kumar Adiga

**Affiliations:** 1 Centre of Excellence in Clinical Embryology, Manipal Academy of Higher Education, Manipal, India; 2 Division of Reproductive Genetics, Manipal Academy of Higher Education, Manipal, India; 3 Division of Reproductive Biology, Department of Reproductive Science, Kasturba Medical College, Manipal Academy of Higher Education, Manipal, India

**Keywords:** assisted reproductive technology, fertility care, India, infertility, LMIC

## Abstract

Historically, infertility has been stigmatized in Indian society, primarily due to societal norms that equate marriage with procreation. In the twentieth century, India focused primarily on over-fertility in its family planning programs, with little attention given to the complexities of infertility. The introduction of assisted reproductive technology (ART) in the late 1970s marked a global revolution, including in India, offering hope to infertile couples. Despite a significant rise in ART clinics offering a wide range of treatment options in recent years, challenges remain, particularly regarding affordability. In India, ART is typically dominated by the private sector as government support remains limited. Efforts to standardize ART practices, including the establishment of the National ART & Surrogacy Registry and the ART Act aim to regulate, improve outcomes, and curb unethical practices. Despite these advancements, the high cost of treatment cycles and lack of insurance coverage limit couples' ability to undergo fertility treatment. Addressing these issues requires a multifaceted approach, including policy reform, increased public awareness, and the development of affordable treatment options to ensure broader access to reproductive care across India.

**Lay summary:**

India has seen a significant rise in infertility clinics, predominantly in the private sector, catering to the need for increased infertility rates. With regulatory policies enforced by the government, standards for the establishment and functioning of assisted reproduction clinics have been prescribed. However, accessibility and affordability of these services remain a question for many infertile couples, especially in rural areas, as infertility treatment costs are high and are not subsidized by the government. Strategies are required to address the financial challenges as well as the shortage of qualified professionals in the assisted reproduction industry. In this review, we chart the development of assisted reproduction in India, with a focus on the regulatory policies and the current challenges that prevent broader access to reproductive care.

## Historical background on fertility issues in India

Infertility has long been shrouded in stigma and misunderstanding, particularly in conservative societies where childbearing is closely tied to social status and family expectations. In India, where traditional views on family and reproduction are deeply rooted, infertility often carries a heavy social burden. The historical perspective on fertility issues in India provides insights into how these problems have been perceived, addressed, and managed over time (Rao 2004, Sharma *et al*. 2018). Until the beginning of the 21st century, not much attention was given to infertility management, and there was a lack of scientific information on understanding the trends and consequences of infertility in India. Historically, family planning programs were viewed exclusively as the patterns and determinants of over-fertility rather than infertility (Sarkar & Gupta 2016).

In India, the estimated prevalence of infertility is approximately 3–14% (Ganguly & Unisa 2010, Sarkar & Gupta 2016). The Indian Council of Medical Research (ICMR) had earlier suggested that approximately 20 million couples are likely to be infertile in India at any given time (ICMR & NAMS 2005). Since parenthood is given much significance and is considered the primary goal of marriage in Indian society, any delay in conception among married couples leads to stigmatization and harassment by the family, thereby causing a negative influence on the couple’s mental and emotional health (De *et al*. 2017). Further, infertility has been associated with a poor quality of life, marital discord, anxiety, depression, sexual dysfunction, and other psychosocial consequences (Dillu *et al*. 2013, Jisha & Thomas 2016, Bose *et al*. 2021).

On 3 October 1978, the world’s second and India’s first *in vitro* fertilization (IVF) baby, Kanupriya, was born through the efforts of Subhas Mukherjee and his colleagues in Kolkata. Mukherjee used gonadotropins for ovarian stimulation, harvested oocytes through the transvaginal route by colpotomy, and performed frozen-thawed embryo transfer into the uterus (Kumar 1997). Subsequently, the *in vitro* fertilization and embryo transfer (IVF-ET) and gamete intrafallopian transfer (GIFT) program was launched at the Institute for Research in Reproduction and the King Edward Memorial Hospital, Bombay, India (Kumar *et al*. 1988).

### Demographic factors influencing infertility

Several demographic factors influence infertility rates in India, a country with diverse backgrounds, varied quality of life, and accessibility to healthcare systems, as well as different climatic conditions. The fertility rate in India has seen a decline from 4.60 (1980) to 1.91 (2021) and is projected to decline further to 1.29 by 2050 (GBD 2021 Fertility & Forecasting Collaborators 2024). Further, there is a shift in the demographic trend of advanced-age women seeking fertility care due to social reasons such as delayed marriage, higher contraceptive use, and career focus (Ganguly & Unisa 2010, Syamala 2012, Kundu *et al*. 2023). The increased prevalence of polycystic ovarian syndrome (PCOS), obesity, and sexually transmitted infections among younger women is affecting fertility (Shah 2017). Interestingly, it has been suggested that the ovarian reserve of Indian women is poorer compared to Caucasian women, which may affect the outcomes of infertility treatment (Iglesias *et al*. 2014).

### Seeking ART in the Indian context

Providing assisted reproductive technology (ART) services in a low- or middle-income country (LMIC) necessitates understanding the country-specific extent and nature of infertility issues, along with identifying pre-existing resources that can be utilized (Sharma *et al*. 2009, Bahamondes & Makuch 2014). In some LMICs, apart from affordable and accessible ART services, the availability of qualified skilled personnel is lacking, possibly due to the high treatment costs, and inadequate infrastructure, along with cultural, religious, and legal barriers. In Indian society, where fertility is so highly valued that womanhood is often equated with motherhood, ART offers hope to those struggling with infertility, though affordability remains an issue. Couples from higher socioeconomic backgrounds, seeking to have their own biological children, can now utilize ART services. However, couples with low socioeconomic status are deeply concerned about the cost incurred and the success rate of the treatment (Ga & Muvvala 2023, Njagi *et al*. 2023).

The huge disparity in access to ART treatment across the world reflects global inequalities in reproductive health and the position of women in society (Jain 2006, Marmot *et al*. 2010, Chambers *et al*. 2013). However, infertile couples in India have access to a wide range of fertility treatment options as shown in Table 1. The use of donor gametes is available to couples if there is a medical indication. However, centers cannot share surplus embryos from couples undergoing treatment with other recipients. The PCPNDT (pre-conception-pre-natal-diagnostic-techniques) act prohibits ART clinics from providing couples with a child of predetermined sex except for preventing or treating sex-linked disorders. Commercial surrogacy is prohibited whereas altruistic surrogacy is permitted only to Indian citizens (Gazette of India, ART (Regulation) Act 2021).

**Table 1:** Fertility treatment options available at ART centres in India and their extent of utility.

Category/ART services	Extent of utility of services* (low to high)
IVF/ICSI	
Conventional IVF-ET	●●●⚪⚪
ICSI-ET	●●●●●
Blastocyst culture	●●●⚪⚪
Frozen embryo transfer (FET)	●●●●⚪
Testicular sperm extraction/aspiration	●●●⚪⚪
Third party reproduction	
Donor oocyte	●●●●⚪
Donor sperm	●●●●⚪
Surrogacy	●●●⚪⚪
Mitochondrial transfer	⚪⚪⚪⚪⚪
Fertility preservation	
Sperm freezing	●●●●●
Oocyte freezing	●●●●⚪
Embryo freezing	●●●●●
Gonadal tissue freezing	●⚪⚪⚪⚪
Add-ons in ART	
Assisted hatching/ EmbryoGlue	●●●●⚪
Oocyte activation	●●●⚪⚪
PGT-A	●●●⚪⚪
PGT-M/PGT-SR	●●●⚪⚪
Microfluidics	●●⚪⚪⚪
Time-lapse embryo culture	●●●⚪⚪
IMSI	●⚪⚪⚪⚪
AI-ML	●●⚪⚪⚪
PICSI	●●⚪⚪⚪
*In vitro* maturation of oocytes	●⚪⚪⚪⚪
ERA	●●●⚪⚪

*Based on services offered on the websites of leading ART centers in India.

AI-ML, artificial intelligence combined with machine learning; ERA, endometrial receptivity assay; ET, embryo transfer; ICSI, intracytoplasmic sperm injection; IMSI, intracytoplasmic morphologically selected sperm injection; PGT, preimplantation genetic testing (A-Aneuploidy, M-Monogenic disorders, SR-Structural rearrangements); PICSI, physiological ICSI.

**Figure 1:**
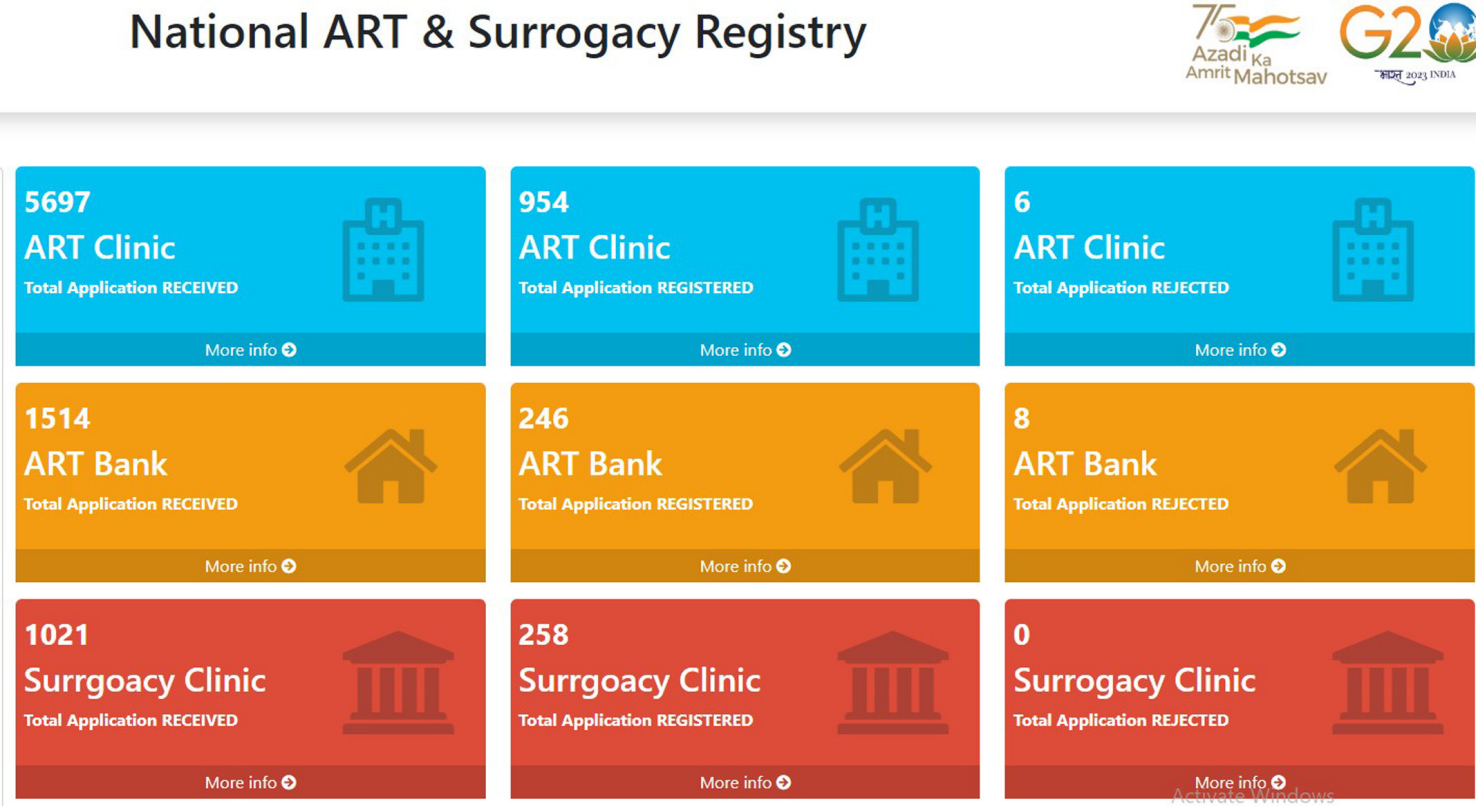
The number of assisted reproductive technology facilities available in India (*Source*: https://artsurrogacy.gov.in/; last accessed 1 August 2024).

The rising infertility rates have seen a proportional increase in ART clinics, both in the private and public sectors, across the country. Over the last two decades, India has been witnessing significant growth in the number of centers offering assisted reproductive technology services. The National ART and Surrogacy Registry is an online public record system of ART clinics/banks and surrogacy clinics in India (http://registry.artsurrogacy.gov.in). The national registry acts as a central database of all the ART clinics, banks, and surrogacy clinics in the country through which the details of all the clinics and banks in the country, including the nature and types of services provided by them, outcomes of the services and other relevant information, can be obtained. The data generated from the national registry is being used for making policies and guidelines, and in identifying potential research areas in India. In 2010, India had about 500 ART centers, whereas in 2019, the number raised to 1500 (IFFS Surveillance 2019). The number of various assisted reproductive facilities available in India as of 1 August 2024 is shown in Fig. 1.

Earlier, the public health system in the country did not offer adequate preventive, curative, and counseling services for infertility. Therefore, most people preferred opting for infertility services in the private sector (Widge & Cleland 2009). Military personnel in India are provided with free IVF treatment through regional military hospitals (Ga & Muvvala 2023). On the other hand, India now has more than 5000 centers in the private sector out of which 15–20 large corporate chain centers are providing ART services across the country. This is a significant moment in providing ART access to needy couples. Though the fertility industry in India was an integral part of the country’s growing medical tourism industry, which experienced about 30% growth in 2000 (Tattara 2010), but due to regulatory restrictions imposed on surrogacy, a decline has been seen in this sector (Pal 2023). The Surrogacy (Regulation) Act 2021, while aimed at regulating surrogacy in India, excludes a segment of the population such as unmarried couples or individuals and same-sex couples (Ministry of Law and Justice 2021) (Table 2). Also, restricting the surrogacy services to only Indian citizens or permanent residents of India, and the introduction of altruistic surrogacy with a lack of clarity on compensation of services, could be contributing factors to this decline (Kashyap & Tripathi 2023, Pal 2023).

**Table 2:** Overview of the regulatory policies governing ART and surrogacy services in India.

	ART (Regulation) Act 2021*	Surrogacy (Regulation) Act 2021^
Objectives of the regulation	Implement legislation in ART. Regulate functioning of ART clinics and ART banks. Prevent unethical or exploitative practices in ART. Safeguard rights of infertile couples/women undergoing infertility treatment. Protection of children born through ART.	Establishing criteria for legal surrogacy. Regulation of surrogacy services. Safeguard rights of the surrogate. Prevention of child abandonment. Prohibition of commercial surrogacy.
Eligibility criteria	Infertile couple: married infertile couples where the woman is between 21 and 50 years of age and the man between 21 and 55 years of age. Single women between 21 and 50 years of age. Oocyte donor: Married woman of 23–35 years of age, with an existing child of her own. Sperm donor: 21–55 years of age.	Commissioning couple: intending infertile couples of Indian nationality married for at least 5 years, aged between 26 and 55 years for the male and 23 and 50 years for the female, with proven infertility and no surviving healthy children. Commissioning single women: intending woman between 35 and 45 years of age, who is a widow or divorcee. Surrogate mother: woman of 25–35 years, married at least once in her lifetime, with a surviving child, and is a close relative of the commissioning couple.
Pros of the regulation	Guidelines on duties of healthcare professionals, functioning of ART clinics/banks prescribed. Establishment of a national registry Financial security for oocyte donors. Inclusion of pre-implantation genetic testing & fertility preservation services. IVF access to couples with HIV. Addresses conducting of research on human gametes/embryos. Legitimacy of children born through ART services.	Curb exploitation of surrogate mothers. Insurance coverage for surrogate mothers for 16 months. Legitimacy of children born out of surrogacy.
Cons of the regulation	Exclusion of services to live-in couples, same-sex couples, unmarried/divorced/widowed men, LGBTQ+ individuals. High registration fees for ART centers. Only onetime oocyte donation in the lifetime of a donor.	Exclusion of services to live-in couples, same-sex couples, single male parents, LGBTQ+ individuals. Exclusion of services to intending parents with a surviving child.
Issues to be addressed	Compliance of registration of all ART clinics across India. Maintenance of national registry, confidentiality of information as well as regulations for the use of registry data. Regulations for foreign citizens to avail ART treatment in India. Regulate cost of ART services.	Clarity on surrogacy contracts. Effectiveness of altruistic surrogacy in the context of the Indian familial system needs assessment. Child welfare in altruistic surrogacy when the surrogate is a close member of the family.

*Gazette of India, ART (Regulation) Act (2021), Jamwal & Yadav (2023), Manisha *et al.* (2024); ^Ministry of Law and Justice (2021), Kashyap & Tripathi (2023), Garg *et al. (*2023).

Most Indian ART clinics have started using advanced technologies such as preimplantation genetic testing, artificial intelligence, endometrial receptivity assay, and microfluidics in their programs despite the lack of evidence for most add-ons in IVF (Mahajan 2015, Selvaraj *et al*. 2016, Kotdawala *et al*. 2019, Patil *et al*. 2019, Nayar *et al*. 2022, Srinivas *et al*. 2022, ESHRE Add-ons working group 2023, Lundin *et al*. 2023). Guidelines have been provided by expert professionals to regulate the practice of using add-ons in ART cycles (Malhotra *et al*. 2021). The availability of specialized techniques like ovarian tissue cryopreservation is limited to a few centers (Salama *et al*. 2020). On the other hand, non-invasive metabolomic screening of embryos (Jijo *et al*. 2022), and immature testicular tissue cryopreservation from pediatric patients needing oncological therapy are being used under research settings (Tholeti *et al*. 2024).

One of the major obstacles to successfully establishing ART centers in LMICs is the shortage of qualified human resources such as fertility clinicians, embryologists, nurses, social workers, and laboratory technicians (Ombelet & Lopes 2024). However, Indian higher education institutes have introduced postgraduate programs in clinical embryology and reproductive medicine specialities, which is a significant milestone in capacity building. As of now, eleven higher education institutes offer super speciality training for fertility clinicians, though the number of intakes is restricted to 23 in total (https://www.nmc.org.in accessed on 28 September 2024). In contrast, more than 30 institutes offer postgraduate-level training in clinical embryology with an annual intake of approximately 300 trainees. However, doctoral and postdoctoral level training in embryology is restricted to very few universities (www.manipal.edu accessed on 28 September 2024). On the other hand, there are no training programs available for nurses and counselors who are also integral parts of the ART program.

### Regulatory mechanisms

The National ART and Surrogacy Board was constituted on 4 May 2022, (Govt of India notification no CG-DL-E-04052022-235539 2022), which is a central body for advising the central government on policy matters on ART, reviewing and monitoring the implementation of the rules and regulations; laying down a code of conduct for the personnel working at the ART facilities; setting minimum standards of laboratory, physical infrastructure, diagnostic equipment, and carrying other functions as prescribed. Indian ART clinics are allowed to provide services to women above 21 years and below 50 years of age and men above 21 years and <55 years of age (Table 2).

While the ART act aims to regulate fertility services in the country, it is still in its preliminary state of implementation. The act has restricted single fathers, same-sex couples, and unmarried couples from availing themselves of ART treatment thereby curbing their desire for parenthood (Table 2). It is hoped that strict implementation of the act is crucial to safeguard the interests of infertile couples, gamete donors, and children born through these procedures.

### Financial implications

In India, ART is available for couples but is not subsidized by the government. Though very few public hospitals offer ART treatment for free or at a subsidized cost, this cannot meet the country’s demand. Earlier, the public sector in infertility management did not meet patients’ expectations due to factors such as limited service, inadequate infrastructure and management, and lack of expert professionals and protocols, to name a few (Widge & Cleland 2009). The state government of Goa was one of the first to introduce initiatives to provide financial assistance to tribal couples suffering from infertility (Velip 2015, Times News Network 2016). Goa further introduced an initiative to offer free ART treatment at public hospitals to infertile couples (Shetye 2023). Other states like Rajasthan and Maharashtra have also started to place their focus on areas such as infertility treatments with an aim to provide financial support to infertile couples (Perappadan 2023, Chakraborty 2024).

The cost incurred to undergo one ART cycle is higher than the average annual income of a patient (166.4%) (Njagi *et al*. 2023). In spite of the financial constraints, the majority of the couples agreeing to the first IVF cycle had to stop treatment when repeated attempts of ART cycles were indicated (Kulkarni *et al*. 2014). There have been initiatives taken by a few centers to make the services affordable by reducing the costs of stimulation, diagnostics, and IVF procedures without compromising on quality (Aleyamma *et al*. 2011). It was suggested that the use of simplified IVF culture systems, as part of the ‘Walking Egg’ project or use of INVOcell device, are effective low-cost strategies to bring about affordability and higher accessibility to infertility treatments in LMICs (Ombelet 2013, Chiware *et al*. 2021, Ombelet *et al.* 2022*a*, *b*). Similarly, standardized investigation of the couple at minimal costs under a ‘one-stop diagnostic clinic’ can reduce the treatment cost (Ombelet *et al*. 2012). However, there is no scientific data on how these approaches have been implemented and benefited infertile couples in India.

### Challenges

Despite the positive trends, challenges remain in India’s ART sector. The cost of a treatment cycle can be prohibitive for most couples, particularly in rural India where access to advanced medical facilities is limited. High treatment costs and limited success rates limit the patients’ ability to undergo multiple treatment attempts. Also, health insurance policies in India typically do not cover infertility treatments, making it difficult for couples to afford these services. Recently, a few insurance companies have begun to offer health insurance plans to cover infertility treatments, especially to employees in the corporate sector; however, the riders attached to the coverage are limiting access to treatment (Sarkar & Basu 2023, Sinha 2024, Singh 2024). There is still a need for continued efforts to combat the deep-seated stigma associated with infertility.

It has been suggested to have more rigorous data reporting, collection, and verification from LMICs to enable a meta-analysis in the future (Kushnir *et al*. 2017). Also, research in the development of indigenous products such as hormones, culture products, and disposables can reduce the treatment cost significantly, thereby increasing the number of patients receiving the treatment. Addressing these challenges requires a multi-faceted approach involving policy changes, increasing public awareness, enhancing the accessibility and affordability of healthcare services, maintaining a good research ecosystem, and improving the regulatory environment.

## Conclusion and future perspectives

India's journey toward accepting ART as a legitimate and respectable solution to infertility management is a testament to the power of education, technology, and changing societal norms. As the conversation around infertility continues to evolve, it is hoped that more couples will feel empowered to seek the help they need, free from stigma and judgment. By embracing ART, India is not only helping individuals fulfill their dreams of parenthood but also paving the way for a more understanding and supportive society. However, the development of more affordable and accessible ART services will improve overall access to reproductive care in India.

## Declaration of interest

The authors declare that there is no conflict of interest that could be perceived as prejudicing the impartiality of this review.

## Funding

This work did not receive any specific grant from any funding agency in the public, commercial, or not-for-profit sector.

## Author contribution statement

SKA and PT conceived, organized, and wrote this review. SU and GPK reviewed the manuscript and provided critical comments.
